# Astrocytes in *Atp1a2*‐deficient heterozygous mice exhibit hyperactivity after induction of cortical spreading depression

**DOI:** 10.1002/2211-5463.12848

**Published:** 2020-04-23

**Authors:** Hiroki Sugimoto, Masaaki Sato, Junichi Nakai, Kiyoshi Kawakami

**Affiliations:** ^1^ Division of Biology Center for Molecular Medicine Jichi Medical University Tochigi Japan; ^2^ Graduate School of Science and Engineering Saitama University Japan; ^3^ Brain and Body System Science Institute Saitama University Japan; ^4^ Laboratory for Mental Biology RIKEN Center for Brain Science Saitama Japan; ^5^ Present address: Graduate School of Dentistry Tohoku University Miyagi 980‐8575 Japan

**Keywords:** astrocyte, *Atp1a2*, calcium imaging, CSD, FHM2, G‐CaMP7, migraine

## Abstract

The *ATP1A2* coding α2 subunit of Na,K‐ATPase, which is predominantly located in astrocytes, is a causative gene of familial hemiplegic migraine type 2 (FHM2). FHM2 model mice (*Atp1a2^tmCKwk/+^*) are susceptible to cortical spreading depression (CSD), which is profoundly related to migraine aura and headache. However, astrocytic properties during CSD have not been examined in FHM2 model mice. Using *Atp1a2^tmCKwk/+^* crossed with transgenic mice expressing G‐CaMP7 in cortical neurons and astrocytes (*Atp1a2^+/−^*), we analyzed the changes in Ca^2+^ concentrations during CSD. The propagation speed of Ca^2+^ waves and the percentages of astrocytes with elevated Ca^2+^ concentrations in *Atp1a2^+/−^* were higher than those in wild‐type mice. Increased percentages of astrocytes with elevated Ca^2+^ concentrations in *Atp1a2^+/−^* may contribute to FHM2 pathophysiology.

## Graphical abstract

*Atp1a2* *^+/^* *^−^* is a model mouse of familial hemiplegic migraine type 2. The numbers of astrocytes with elevated Ca^2+^ concentrations in *Atp1a2* *^+/^* *^−^* (11.0 ± 2.8 astrocytes) were larger than those in wild‐type mice (5.7 ± 2.5 astrocytes) after induction of cortical spreading depression (which is closely associated with migraine) by application of KCl.

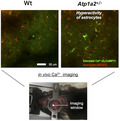

**Abbreviations**
CSD: cortical spreading depression
FHM: familial hemiplegic migraine

Migraine is a common disorder characterized by recurrent debilitating headache attacks [1] that are preceded by aura in a third of patients [2]. Cortical spreading depression (CSD) is a propagating depolarizing wave in neurons and glial cells that spreads across the cerebral cortex and that is followed by a subsequent sustained suppression of spontaneous neuronal activity [3, 4]. Migraine patients show multiphasic cerebrovascular changes that are often observed in CSD during visual aura. Multiphasic cerebrovascular changes appear to be directly linked to the aura percept in both space (retinotopy) and time [5]. In addition, migraine headache depends on the activation of the trigeminovascular system [4, 6]. Animal experiments have indicated that CSD activates meningeal nociceptors in the trigeminovascular system [7]. These results indicate that CSD is closely associated with migraine aura and headache [4].

Findings from the monogenic form of diseases provide clear insights into the molecular pathways of diseases. Familial hemiplegic migraine (FHM) is a rare monogenic form of migraine with aura including motor weakness [1], and three FHM causative genes have been identified: *CACNA1A* (coding for the α1a subunit of the Ca_V_2.1 calcium channel) for the FHM1 [8]; *ATP1A2* (coding for the α2 subunit of Na,K‐ATPase) for the FHM2 [9]; and *SCN1A* (coding for the α subunit of the Na_V_1.1 sodium channel) for the FHM3 [10]. Gain‐of‐function mutations in the *CACNA1A* gene causing FHM1 lead to increased Ca^2+^ influx in presynaptic terminals, which, in turn, results in increased glutamate release in the synaptic cleft [11, 12]. Loss‐of‐function mutations in *ATP1A2* causing FHM2 lead to the reduced clearance of glutamate and K^+^ by astrocytes [13]. Loss‐of‐function mutations in *SCN1A* causing FHM3 lead to the reduced inhibitory activity of inhibitory interneurons, which results in increased excitatory neuronal activity [14]. Increased glutamate in the synaptic cleft results in increased cortical excitatory neurotransmission [10, 15]. Consistently, a decreased threshold for CSD induction has been observed in FHM1 and FHM2 model animals [16, 17].

Na,K‐ATPase consists of α and β subunits and maintains the electrochemical gradient of Na^+^ and K^+^ across the cell membrane using the energy of ATP hydrolysis [18, 19]. There are four isoforms of the α subunit (α1–α4) and three isoforms of the β subunit (β1–β3). The α2 subunit, which is coded by *Atp1a2,* is predominantly localized in astrocytes in adult rats [20]. Heterozygous *Atp1a2* knockout mice, *Atp1a2^tmCKwk/+^* [21], and heterozygous knock‐in mice carrying the human W887R mutation in *Atp1a2* [17] have been developed for FHM2 model animals. CSD was induced by electric stimulation or KCl application into the cortex of these animals, and both types of mice showed a decreased threshold for CSD induction than wild‐type (wt) mice, suggesting increased CSD susceptibility [17, 22]. In addition, *Atp1a2^tmCKwk/+^* mice showed enhanced fear/anxiety behaviors [21] and obesity by hyperphagia [23]. These observations are consistent with the characteristics of FHM2 patients showing anxiety [24, 25] and obesity [26], indicating that the *Atp1a2^tmCKwk/+^* mouse reproduces FHM2 symptoms and is a useful model animal for clarifying the pathophysiology of FHM2. However, the properties of astrocytes and their possible influence on neuronal activity during CSD have not been examined in FHM2 model mice.

To this end, we analyzed changes in Ca^2+^ concentrations using the fluorescent calcium indicator G‐CaMP7 [27] in astrocytes and neurons as a proxy for their excitability [28, 29, 30]. *Atp1a2^tmCKwk/+^* mice crossed with transgenic mice expressing G‐CaMP7 in the cortex and hippocampus [31] were used to monitor changes in Ca^2+^ concentration in cortical neurons and astrocytes.

## Materials and methods

### Ethics statement for the animal experiments

All animal experiments were carried out in a humane manner. The Institutional Animal Experiment Committees of Jichi Medical University and the Saitama University approved this study. This study was conducted in accordance with the Institutional Regulations for Animal Experiments and Fundamental Guidelines for Proper Conduct of Animal Experiments and Related Activities in Academic Research Institutions under the jurisdiction of the MEXT of Japan.

### Animals

*Atp1a2*‐deficient mice (*Atp1a2^tmCKwk/+^*, [21]) and G7NG817 transgenic mice [31] expressing the fluorescent calcium indicator G‐CaMP7 [27] were crossed. Mice of the F1 generation (*Atp1a2^tmCKwk/+^*; G7NG817^+/−^) were inbred. Then, *Atp1a2^tmCKwk/+^*; G7NG817^+/+^ and *Atp1a2^+/+^*; G7NG817^+/+^ mice were selected from the F2 generation and crossed. In the F3 generation, female *Atp1a2^tmCKwk/+^*; G7NG817^+/+^ and *Atp1a2^+/+^*; G7NG817^+/+^ mice were called *Atp1a2^+/−^* and wt mice, respectively, and were used in this study. Mice were housed under a 12‐h light/dark cycle (lights on from 7:00 AM to 7:00 PM) in a temperature‐controlled room (22 °C ± 2 °C). Food and water were provided *ad libitum*.

### Surgical procedure

**Fig. 1:**
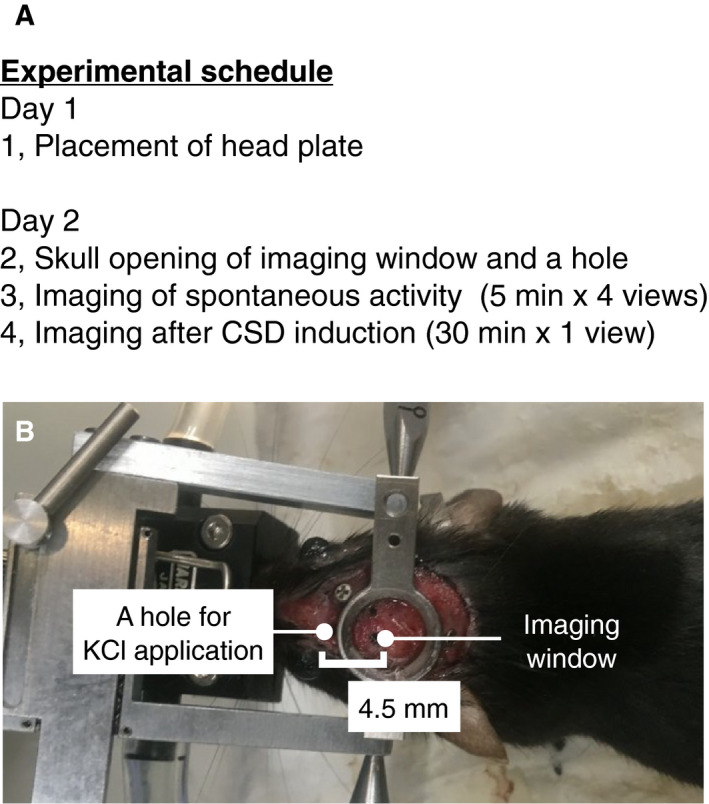
Experimental schedule. (A) Experimental schedule. (B) Head plate‐attached mouse.

Female postnatal 2‐ to 5‐month‐old mice were used (Fig. 1A). On day 1, mice were anesthetized with isoflurane (3% induction, 1.5% maintenance) and placed in a stereotaxic frame with ear bars [32]. After skull exposure, a stainless steel head plate with a circular opening (7 mm diameter) was placed over the left parietal bone and was attached to the skull with dental acrylic. The mice were returned to their home cages.

On day 2, the mice were anesthetized with isoflurane (3% induction, 1.5% maintenance) supplemented with chlorprothixene (1 mg·kg^−1^, i.p.). Atropine (0.3 mg·kg^−1^, s.c.) and dexamethasone (2 mg·kg^−1^, s.c.) were administered to reduce respiratory secretions and brain edema, respectively [33]. The mice were placed in a stereotaxic frame via the head plates (Fig. 1B). A piece of skull (~ 2 mm diameter) within the circular opening of the head plate was surgically removed for imaging. Then, a small hole (~ 1 mm in diameter) was opened 4.5 mm anterior to the center of the imaging window for KCl application. Ten microliters of 100 μm sulforhodamine 101 was applied to the exposed cortical surface to label astrocytes. After 5 min of application, the cortical surface was washed 5 times with cortical buffer (123 mm NaCl, 5 mm KCl, 10 mm glucose, 2 mm CaCl_2_, 2 mm MgCl_2_, and 10 mm HEPES at pH 7.4) and covered with 1% agarose.

### *In vivo* two‐photon imaging

During two‐photon imaging, the mice were anesthetized with 1% isoflurane. Body temperature was maintained with a heating pad throughout the imaging session. To avoid the risk of evoking abnormal cortical neural activity by sulforhodamine 101 [34], images were acquired using a Nikon A1RMP microscope equipped with an Apo LWD 25 × 1.10 objective (Nikon, Tokyo, Japan) after about 60 min from application of sulforhodamine 101. For imaging spontaneous activity, 512 × 512 pixel images (field size 254 × 254 µm at depths of 200 µm and 250 µm) were acquired at 15 frames per second for 5 min. From each mouse, two fields of view were imaged each at 200 µm and 250 µm deep.

Cortical spreading depression was induced after imaging spontaneous activity. Imaging was started immediately after the application of 2 μL of 1 m KCl to the hole. Images in 512 × 512 pixels (field size 508 × 508 µm) were acquired at one frame/ 3 s at a depth of 200 µm for 30 min.

### Data analysis

#### Analysis of the spontaneous activity of neurons

**Fig. 2:**
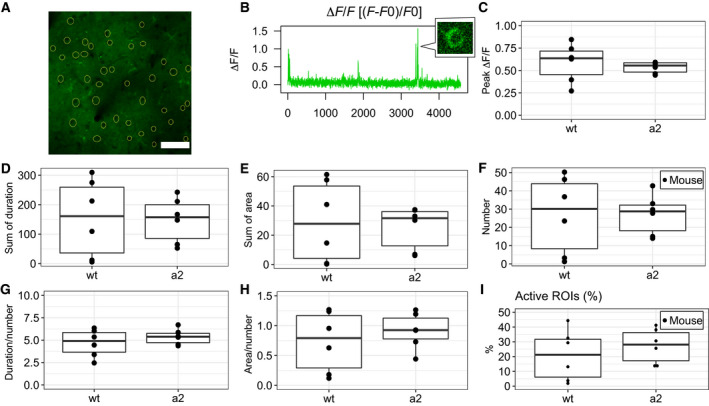
Spontaneous activity of neurons in the cortex. (A) Image of the cortex at a depth of 200 μm. Manually selected ROIs (yellow circles). Bar = 100 μm. (B) Data of Δ*F*/*F* calculated by (*F* − *F*0)/*F*0. A small window shows cells with increased fluorescence. (C) Peak ∆*F*/*F* of activity. (D) Sum of the duration of activity. (E) Sum of the area of activity. (F) Number of activities. (G) Duration divided by the number of activities. (H) Area divided by the number of activities. (I) Percentage of active ROIs. C‐I were not significantly different between wt mice (*N* = 6) and *Atp1a2^+/−^* mice (a2, *N* = 6).

We defined regions of interest (ROI) on neurons manually from average Z‐stacked images (Fig. 2A), and the mean values (*F*) of fluorescence intensity within the ROIs in each frame of the time‐lapse image sequences were calculated (Fig. S1A) by fiji (ImageJ) (National Institutes of Health, Bethesda, MD, USA) software [35]. The obtained data were subsequently analyzed by r software [36]. The baseline value (*F*0) of each ROI was defined as the first quartile of all the data points. *F*0 was used to calculate Δ*F*/*F* as (*F*‐*F*0)/*F*0 (Fig. 2B). A simple moving average with a window size of five frames was applied to Δ*F*/*F* (Fig. S1B). Neuronal activity was defined as the local maxima of Δ*F*/*F* above the threshold (4.5 SD from *F*0; Fig. S1C,D). Then, the duration, area, number, and peak of activities were measured (Fig. S1E‐G).

#### Analysis of the activities of neurons and astrocytes during CSD

**Fig. 3:**
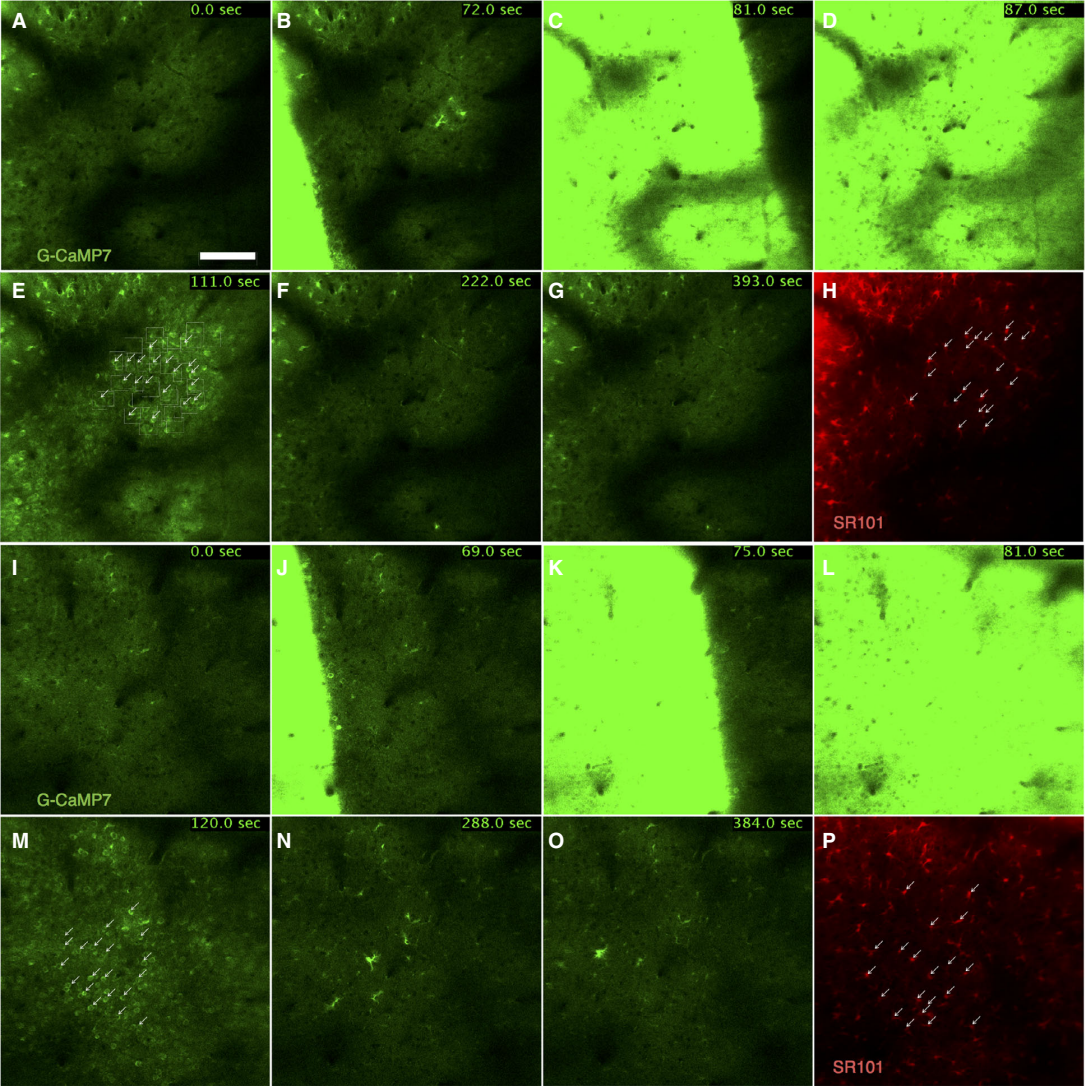
Representative time‐lapse image sequence after CSD induction. Time‐lapse image sequence of G‐CaMP7 fluorescence intensity of wt mice (A–G) and *Atp1a2^+/−^* mice (I–O) after CSD induction. Duration indicates time after imaging start. Bar = 100 μm. (E, M) Arrows indicate neurons selected as ROIs. (H, P) Image labeled by the astrocyte marker sulforhodamine 101 (SR101). Arrows indicate astrocytes selected as ROIs.

We identified ROIs on 20 neurons and 20 sulforhodamine 101‐labeled astrocytes selected manually from images after propagating waves of transiently increased G‐CaMP7 fluorescence (Fig. 3E,M and H,P). The mean values (*F*) of fluorescence intensity within each ROI in each frame of the time‐lapse image sequences were obtained by fiji software (Fig. S2A). To determine the baseline value, Ca^2+^ waves were defined as the periods during which the mean value of F across 40 ROIs was above 2500 (Fig. S2B,C). Then, the baseline value (*F*0) was defined as the median value of fluorescence intensity before the first Ca^2+^ waves (Fig. S2D). *F*0 was used to calculate ∆*F*/*F* as (*F*‐*F*0)/*F*0 (Fig. S2E). The number of Ca^2+^ waves in each image sequence was counted visually as the number of discontinuous peaks of Ca^2+^ waves (Fig. S2C).

Minimum values of fluorescence intensity were obtained from ΔF/F time series from which increased intensity during Ca^2+^ waves was omitted (Fig. S2F,G). To analyze changes in fluorescence intensity after Ca^2+^ waves, Ca^2+^ waves and 30 frames before and after the Ca^2+^ waves were excluded from the ΔF/F data (Fig. S2H). The rate of change in fluorescence intensity during the periods after (or between) Ca^2+^ waves was quantified as the slope obtained by linear regression (Fig. S2I). The slopes obtained from multiple periods were then averaged for each ROI.

The Δ*F*/*F* time series after or between Ca^2+^ waves generally appeared as upward curves. To evaluate the transient elevation of fluorescence intensity in neurons and astrocytes, the baselines were normalized by calculating Δ*F*/*F* − (slope × frame + intercept). Slope and intercept were calculated by linear regression using the mean values of Δ*F*/*F* from 40 ROIs. Peaks of activity were defined as the local maxima of normalized Δ*F*/*F* values above the threshold (5, 7, and 10 times the SD of fluorescence intensity before the first Ca^2+^ waves; Fig. S2J,K). The number, duration, and peak of activity events for each ROI and fractions of active ROIs among a total of 20 ROIs were calculated.

In each mouse, pairwise correlation of the changes in fluorescence intensity between two different ROIs was calculated using the normalized Δ*F*/*F* data (Fig. 6A,B). The data were binarized according to the presence and absence of activity (1 and 0, respectively) after thresholding (five times the SD of fluorescence intensity before the first Ca^2+^ waves; Fig. 6C). Then, the frames in which all of the ROIs were not active (Fig. 6D) and the ROIs that did not show activity were omitted (Fig. 6E). We then calculated the correlation coefficients between ROIs (Fig. 6F). Correlation coefficients for neuron–neuron, neuron–astrocyte, and astrocyte–astrocyte pairs were separately averaged. To estimate the correlation between changes in fluorescence intensity within single astrocytes, ROIs were manually defined on their cell bodies and processes (Fig. S5A). The mean values (*F*) of fluorescence intensity within each ROI in each frame were then calculated. To determine the baseline value, Ca^2+^ waves were defined as the periods during which the mean value of *F* across all ROIs was above 3700. Then, the baseline value (*F*0) was defined as the median value of fluorescence intensity before the first Ca^2+^ waves. *F*0 was used to calculate Δ*F*/*F* as (*F*‐*F*0)/*F*0. To analyze changes in fluorescence intensity after Ca^2+^ waves, the Ca^2+^ waves and 30 frames before and after the Ca^2+^ waves were excluded from the Δ*F*/*F* data. The baselines were normalized by calculating Δ*F*/*F* − (slope × frame + intercept). The slope and intercept were calculated by linear regression using 10 percentile values from all ROIs. Correlation coefficients of fluorescence changes within single astrocytes were calculated as described above for those between cells.

The speed of propagating waves of transiently increased G‐CaMP7 fluorescence was measured by the distance between the wavefronts of increased fluorescence imaged at nth and (*n* + 1)th frames divided by the time interval between the two frames. The position of the wavefront at the (*n* + 1)th frame was defined as the intersectional position of the perpendicular line from the position at the *n*th frame to the wavefront at the (*n* + 1)th frame. In a wave, we selected three different positions where we could easily draw the perpendicular line, and measured the lengths of three perpendicular lines. Then, the mean values were used for statistical analysis.

### Statistical analysis

All statistical analyses were performed using r [36]. Significance was set at *P* < 0.05. Analysis of variance (ANOVA) was performed to examine the effect of genotype and observation depth. Differences between *Atp1a2^+/−^* and wt mice were tested by *t*‐test or Wilcoxon rank‐sum test.

## Results

### Spontaneous activity

To evaluate whether spontaneous neural activity in FHM2 model mice *Atp1a2^tmCKwk/+^* [21] is different from that in wt mice, we observed spontaneous changes in Ca^2+^ concentrations in the cortex of anesthetized mice by monitoring the fluorescence intensity of the fluorescent calcium indicator G‐CaMP7. *Atp1a2^tmCKwk/+^* and G7NG817 transgenic mice expressing G‐CaMP7 [31] were crossed. Images were obtained from the cortex at depths of 200 µm and 250 µm in *Atp1a2^tmCKwk/+^*; G7NG817^+/+^ (*Atp1a2^+/−^*) and *Atp1a2^+/+^*; G7NG817^+/+^ (wt) mice. Sums of the duration, area, and number of activities in each ROI did not show significant effects of genotype and observation depth in two‐way ANOVA (genotype × depth) (Fig. S1H‐J). Similarly, sums of the duration, sum area, and number of activities in each field of view showed no significant effects in two‐way ANOVA (genotype × depth) (Fig. S1K‐M). Peak ∆*F*/*F* of each activity also showed no significant effects in two‐way ANOVA (genotype × depth) (Fig. S1N). Because there is no effect of depth on spontaneous activity, we summed the duration, area, number, and peak ∆*F*/*F* of activities in each mouse (Fig. 2C‐F). Again, sums of the duration, area, number, and peak ∆*F*/*F* of activities in each mouse were not significantly different between *Atp1a2^+/−^* and wt mice (Fig. 2C‐F). The duration and area divided by the number of activities were similar between *Atp1a2^+/^* ^−^ and wt mice (Fig. 2G,H). The number of active ROIs among all ROIs (40 ROIs) in each mouse (%) was not different between *Atp1a2^+/^* ^−^ and wt mice (Fig. 2I). These observations indicate that spontaneous neural activity did not show any significant differences between *Atp1a2^+/^* ^−^ and wt mice. Spontaneous activity was not observed in astrocytes labeled by sulforhodamine 101 (data not shown) in our experiment, although it has been reported that the G7NG817 transgenic mice show spontaneous activities in astrocytes [31]. The discrepancy may be due to the differences of method of anesthesia and/or the degree of recovery of mice from surgery.

### Activity during CSD

**Fig. 4:**
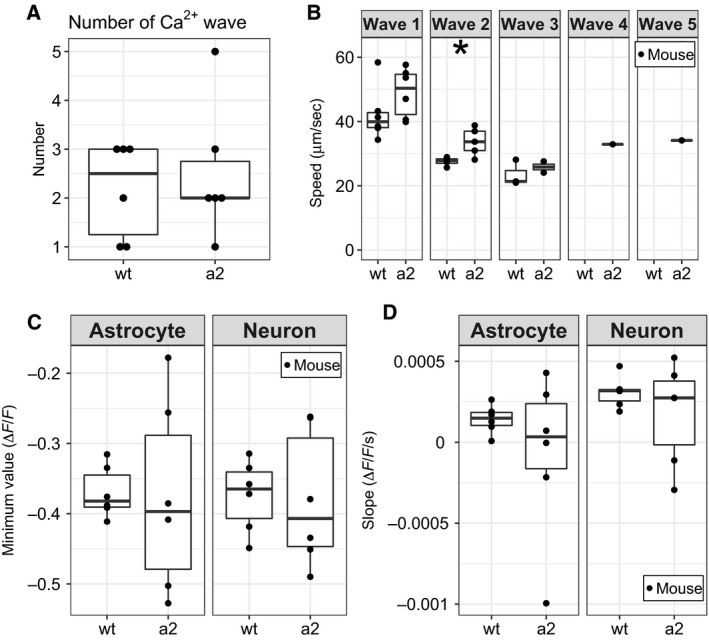
Quantitative data of activity after CSD induction in each mouse. (A) Number of Ca^2+^ waves in wt mice (*N* = 6) and *Atp1a2^+/−^* mice (a2, *N* = 6). (B) Speed of propagating waves of transiently increased G‐CaMP7 fluorescence. The second wave of a2 (*N* = 5) showed significantly faster propagation than that of wt (*N* = 4). (C) The mean minimum value of Δ*F*/*F* and (D) the mean value of the slope of the regression line in wt (*N* = 6) and a2 (*N* = 6) were plotted with astrocytic and neuronal ROIs. *P < 0.05 by *t*‐test.

To examine whether changes in astrocyte properties influence neuronal activity during CSD, we investigated Ca^2+^ concentration changes in astrocytes and neurons during CSD. CSD can be induced by KCl directly applied to the cortex [37]. After the application of KCl, propagating waves of transiently increased G‐CaMP7 fluorescence intensity were observed across the cortex in *Atp1a2^+/−^* (Fig. 3I‐O, Video S2) and wt mice (Fig. 3A‐G, Video S1). Some mice showed multiple propagating waves after a single application of KCl. The number of propagating waves was not different between *Atp1a2^+/^* ^−^ and wt mice (Fig. 4A). However, the speed of the second propagating waves in *Atp1a2^+/−^* mice (33.72 ± 4.33 µm/s) was significantly faster than that in wt mice (27.56 ± 1.38 µm/s; Fig. 4B). This result was consistent with the results showing the faster propagation of CSD in electrophysiological recordings in FHM2 model mice [17, 22]. In electrophysiological recordings*,* propagation speeds of *Atp1a2^tmCKwk/+^* and wt mice were about 4.5 and 3.9 mm/min, respectively [22]. Interestingly, some astrocytes and neurons showed increased fluorescence intensity after the propagation of the waves (Fig. 3N,O). We then quantified the fluorescence intensity in neurons and astrocytes after the propagation of the wave by establishing ROIs on them. Changes in fluorescence intensity in each ROI were quantified as minimum values and slopes calculated by regression analysis. The minimum values of fluorescence intensity (Fig. 4C) and the slopes of fluorescence intensity (Fig. 4D) were similar between *Atp1a2^+/^* ^−^ and wt mice in astrocytes and neurons. In each ROI, minimum values and slopes of fluorescence intensity showed significant differences between *Atp1a2^+/^* ^−^ and wt mice neither in astrocytes nor in neurons (Fig. S3A–D). The results show that the minimum values and slopes of Ca^2+^ concentration changes after the propagation of the wave were similar between *Atp1a2^+/^* ^−^ and wt mice.

**Fig. 5:**
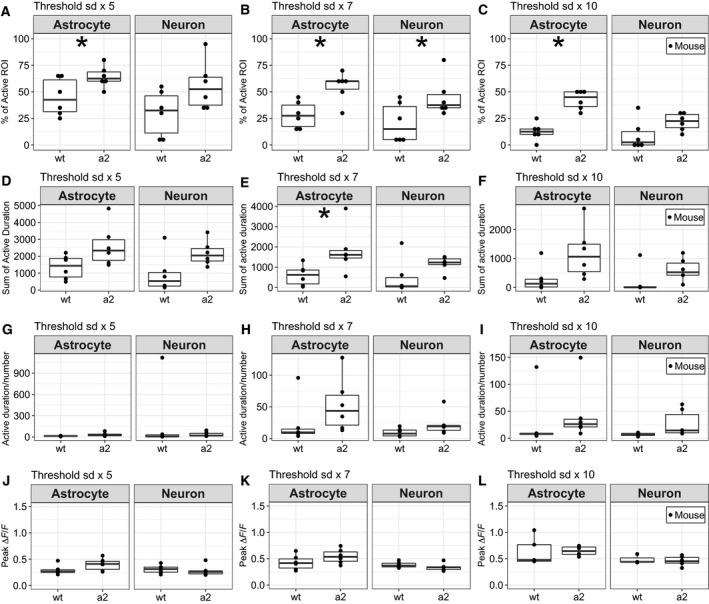
Activity after Ca^2+^ waves in wt mice and *Atp1a2^+/−^* mice (a2). (A) Percentage of active ROIs above the threshold of the SD ×5. (B) Percentage of active ROIs above the threshold of the SD ×7. (C) Percentage of active ROIs above the threshold of the SD ×10. In astrocytes, the percentage of active ROIs in a2 was higher than that of wt at thresholds of the SD ×5, ×7, and ×10. In neurons, the percentage of active ROIs in a2 was higher than that of wt at thresholds of the SD ×7. *P < 0.05 by Wilcoxon rank‐sum test. (D) Sum of the duration of activity above the threshold of the SD ×5. (E) Sum of the duration of activity above the threshold of the SD ×7. In astrocytes, the sum of duration of activity of *a2* was longer than that of wt. *P < 0.05 by *t*‐test. (F) Sum of the activity duration above the threshold of the SD ×10. (G) Activity duration per the number of activities above the threshold of the SD ×5. (H) Duration of activity per the number of activities above the threshold of the SD ×7. (I) Duration of activity per the number of activity above the threshold of the SD ×10. (J) Peak ∆*F*/*F* of activity above the threshold of the SD ×5. (K) Peak ∆*F*/*F* of activity above the threshold of the SD ×7. (L) Peak ∆*F*/*F* of activity above the threshold of the SD ×10. The data of wt (*N* = 6) and a2 (*N* = 6) were plotted with astrocytic and neuronal ROIs.

To evaluate the increase in fluorescence intensity after the propagation of the wave, thresholds were set to 5, 7, and 10 times the SDs of fluorescence intensity before the first Ca^2+^ waves. Activity was defined as fluorescence intensity above these thresholds. For the 5×, 7×, and 10× SD thresholds, *Atp1a2^+/^* ^−^ mice showed significantly higher percentages of active astrocyte ROIs than wt mice (Fig. 5A‐C). The sum of the activity duration of *Atp1a2^+/^* ^−^ in astrocytes was longer than that of wt mice at 7× SD threshold (Fig. 5E). In neurons, *Atp1a2^+/^* ^−^ mice showed significantly higher percentages of active astrocyte ROIs than wt mice at only 7× SD threshold (Fig. 5B). Except for Fig. 5E, there was no difference in the sum of the duration of activity, the duration of activity per the total number of activities, and peak ∆F/F between *Atp1a2^+/^* ^−^ and wt mice at all thresholds (Fig. 5D–L). In each ROI, *Atp1a2^+/^* ^−^ mice showed significantly higher percentages of active ROIs than wt mice at all thresholds (Fig. S4A–C). From these observations, we conclude that more astrocytes in *Atp1a2^+/^* ^−^ mice had higher percentages of cells with increased fluorescence intensity compared with that of astrocytes in wt mice after the propagation of the wave.

**Fig. 6:**
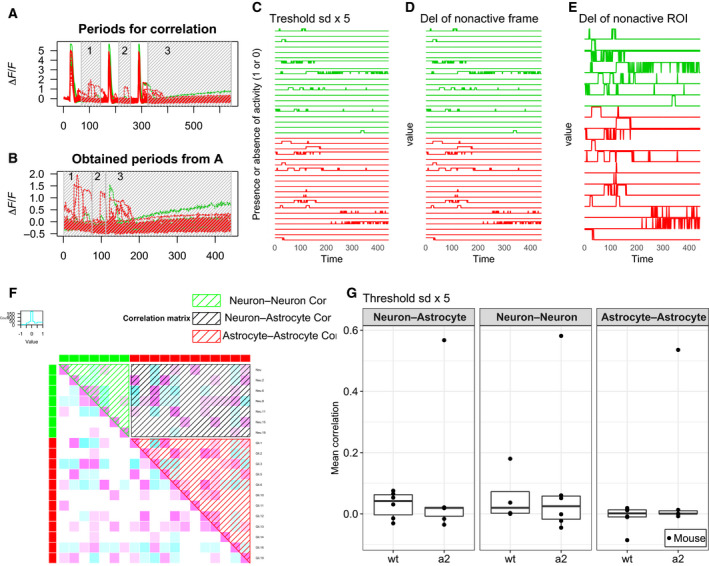
Activity correlation between ROIs. (A) Periods of analysis for activity correlations (gray shadowed box). Green signifies neurons. Red signifies astrocytes. (B) Obtained periods from (A). (C) The data are binarized according to the presence or absence of activity (1 or 0, respectively) from (B) by the threshold of the SD ×5. (D) Deletion of frames in which all ROIs were not active from (C). (E) Deletion of ROIs that did not show activity from (D). (F) Correlation matrix of (E). Green, red, and black shadowed boxes indicate correlation coefficients for neuron–neuron, astrocyte–astrocyte, and neuron–astrocyte pairs, respectively. (G) The mean correlations of wt mice (*N* = 6) and *Atp1a2^+/−^* mice (a2, *N* = 6) in each part of the matrix were plotted.

Finally, to investigate the interaction between astrocytes and neurons, we evaluated synchrony in the changes in fluorescence intensity after Ca^2+^ wave peaks (Fig. 6A,B). In this analysis, Ca^2+^ wave peaks were excluded from the data, and the fluorescence intensity after the peaks in the frames that contained at least one active ROI was analyzed (Fig. 6A–E). We calculated the correlation coefficient for activity between active ROIs, and the correlation coefficients were separately averaged for neuron–neuron, neuron–astrocyte, and astrocyte–astrocyte pairs (Fig. 6F). The mean correlation coefficients were not significantly different between *Atp1a2^+/^* ^−^ and wt mice in neuron–neuron, neuron–astrocyte, and astrocyte–astrocyte pairs in the 5× SD threshold condition (Fig. 6G). We also evaluated the correlation in changes in fluorescence intensity within an astrocyte (Fig. S5A). Although astrocyte processes were not always active when the astrocyte soma was activated, the mean correlation within astrocytes was not different between *Atp1a2^+/^* ^−^ and wt mice (Fig. S5B,C). General anesthesia decreases spontaneous activities of neurons and astrocytes and astrocyte–astrocyte activity correlation compared with those of awake mice [38]. We performed all experiments under isoflurane anesthesia. Therefore, it is not excluded that the activities and the correlation were underestimated.

## Discussion

### Higher propagation speeds in Ca^2+^ waves and increased percentages of cells with elevated Ca^2+^ concentrations in *Atp1a2^+/−^* mice after CSD induction

In this study, CSD was induced by KCl application to the cortex, and then, propagating waves of Ca^2+^ were monitored by G‐CaMP7 fluorescence using GLT‐1‐G‐CaMP7 mice. The second Ca^2+^ waves in *Atp1a2^+/−^* mice had a faster propagation speed than those of wt mice (Fig. 4B), and the percentages of astrocytes that showed elevated Ca^2+^ concentrations after the propagation of Ca^2+^ waves were significantly higher in *Atp1a2^+/−^* mice than in wt mice (Fig. 5A–C). In CSD induction, the propagation of Ca^2+^ waves is shown to occur with CSD propagation, and the propagation speed of the Ca^2+^ wave is similar to that of CSD [39]. Therefore, the difference in the propagation speed of Ca^2+^ waves likely reflects the difference in the propagation speed of CSD. CSD propagation has been proposed to be mediated by the interstitial diffusion of K^+^ or glutamate [40, 41, 42]. Interstitial K^+^ diffusion initiates positive feedback cycles that induce CSD in contiguous dendritic regions, and the clearance of K^+^ and glutamate by astrocytes limits the rate and spatial extent of CSD propagation [39, 42]. Reuptake of glutamate from the synaptic cleft into astrocytes is driven by glutamate transporters GLAST and GLT‐1, utilizing the electrochemical gradient of Na^+^ generated by Na,K‐ATPase [19, 43, 44]. In *Atp1a2^+/−^* mice, when CSD causes a transient increase of glutamate levels in the synaptic cleft, capacity of the glutamate transporters to reuptake the glutamate would become limiting due to the decreased electrochemical gradient of Na^+^. As the result, glutamate levels in the synaptic cleft in *Atp1a2^+/−^* mice are expected to remain higher than those in wt mice. Indeed, mice harboring loss‐of‐function *Atp1a2* mutations show defective glutamate and K^+^ clearance by cortical astrocytes [13]. Taken together, the faster Ca^2+^ wave propagation in *Atp1a2^+/−^* FHM2 model mice is caused by the increased glutamate levels in the interstitial space due to the decreased reuptake of glutamate. It is also known that glutamate promotes increased Ca^2+^ concentrations in astrocytes [45]. Increased Ca^2+^ concentrations after the propagation of Ca^2+^ waves may represent astrocytic reactions to interleukin‐1β (IL‐1β). Studies demonstrate that IL‐1β is released from neurons during CSD [46] and induces transient Ca^2+^ elevations in cultured mouse cortical astrocytes [47].

### Pathophysiology of FHM2

Elevated Ca^2+^ concentrations in astrocytes can trigger the release of gliotransmitters (D‐serine and glutamate), prostaglandin E_2_, and K^+^, which are capable of modulating the function of neighboring glial, neuronal, and vascular cells [48, 49, 50, 51, 52, 53, 54, 55]. In contrast to higher percentages of astrocytes with elevated Ca^2+^ concentrations, we observed little differences in changes in Ca^2+^ concentrations in neurons in *Atp1a2^+/−^* mice (Fig. 5). Furthermore, the correlation of Ca^2+^ concentration changes in neuron–neuron, neuron–astrocyte, and astrocyte–astrocyte pairs (Fig. 6G) was similar between *Atp1a2^+/−^* and wt mice. These results suggest that astrocytes in *Atp1a2^+/−^* mice with elevated Ca^2+^ concentrations may exert modulatory actions on vascular cells without altered astrocyte–neuron coupling. The release of gliotransmitters, prostaglandin E_2_, and K^+^ via elevated Ca^2+^ concentrations in astrocytes could induce vasodilation [53, 54, 55] together with the change in blood flow. Although regional cortical blood flow (CBF) changes during CSD do not differ significantly between *Atp1a2^+/−^* and wt mice [22], increased percentages of astrocytes with elevated Ca^2+^ concentrations may lead to delayed CBF changes, as suggested previously [22]. In addition, cerebral artery vasodilation is frequently associated with prolonged aura in an FHM2 family [56]. Increased percentages of astrocytes with elevated Ca^2+^ concentrations can affect aura symptoms through vasodilation. Because IL‐1β is known to induce elevated Ca^2+^ concentrations in astrocytes [47], the increased percentage of astrocytes with elevated Ca^2+^ concentrations in *Atp1a2^+/−^* mice may represent enhanced inflammatory responses by the IL‐1β‐mediated activation of the trigeminovascular system.

### Astrocytes activation as a common pathophysiology of FHM

It is noted that Ca^2+^ concentration in astrocytes during CSD is higher than that of wt mice in FHM1 model mice, *Cacna1a^R192Q/R192Q^* [57]. Although CACNA1A is predominantly localized in neurons [58], *Cacna1a^R192Q/R192Q^* shows the increased activity of astrocytes as observed in *Atp1a2^+/−^* mice. Therefore, it is likely that the activation of astrocytes causes migraine by a similar mechanism to *Atp1a2^+/−^* mice as described above. Furthermore, FHM3 model mice carrying R1407X mutation in *Scn1a* show the reduced inhibitory activity of inhibitory interneurons, which results in the increased excitatory neuronal activity [14] leading to the increased glutamate levels in the interstitial space. Therefore, it is plausible that the activation of astrocytes occurs by the increased glutamate levels, although this has not been examined. From these, we suggest that the increased activity of astrocytes is a common basis of pathophysiology of three types of FHM, FHM1, FHM2, and FHM3. Astrocytes with elevated Ca^2+^ concentrations, our novel findings in *Atp1a2^+/−^* mice, could cause the migraine aura and headache through vasodilation and inflammatory responses in FHM, although it remains to be elucidated. In future studies, elucidating the role of astrocytes in vasodilation and inflammatory responses during CSD could help reveal the pathophysiology of FHM.

## Conflict of interest

The authors declare no conflict of interest.

## Author contributions

KK and JN supervised the study; HS, MS, and KK designed the experiments; HS and MS performed the experiments; HS analyzed the data and wrote the manuscript; and KK, JN, and MS made manuscript revisions.

## Supporting information


**Fig. S1.** Spontaneous activity of neurons at depths of 200 and 250 μm in the cortex. (A) Representative data of fluorescence intensity *F* obtained from an ROI. First quartile data were used as *F*0. (B) The data applying simple moving average with a window size of 5 frames. (C) Neural activity was defined as the local maxima of Δ*F*/*F* over 4.5 standard deviations from *F*0 (horizontal line). (D) Detection of activity. Red points indicate activity, and black points indicate frames below the threshold. (E–G) The duration (distance between both ends of sequential red points), area (sum of Δ*F*/*F* ‐ threshold in each red point), number, and peak Δ*F*/*F* of activities were measured. (H) Sum of the duration of activity in each ROI. (I) Sum of the area of activity in each ROI. (J) Number of activities in each ROI. ROIs (*N* = 480) were plotted in wild‐type mice (wt) and *Atp1a2^+/−^* mice (a2) at depths of 200 and 250 μm, respectively. (K) Sum of the duration of activity in each field of view. (L) Sum of the area of activity in each field of view. (M) Number of activities in each field of view. Fields of view (*N* = 12) were plotted in wt and a2 at depths of 200 and 250 μm, respectively. (*N*) Peak ∆*F*/*F* of activities (*N* = 294, 351, 317, 332) in wt and a2 at depths of 200 and 250 μm, respectively.

**Fig. S2.** Analysis flow chart after CSD induction. (A) Fluorescence intensity F obtained from 20 neuronal ROIs and 20 astrocytic ROIs. (B, C) Ca^2+^ wave detection. (B) The mean value of fluorescence intensity across 40 ROIs. (C) Ca^2+^ waves (green shadowed boxes) were defined as the periods above 2500 (red horizontal line). (D) The median value of fluorescence intensity before the first wave (pink shadowed box) was defined as *F*0. (E) The data of Δ*F*/*F* calculated by (*F* ‐* F*0)/*F*0. Gray shadowed boxes indicate periods of Ca^2+^ waves. (F) Periods for the analysis of the minimum value (gray shadowed boxes). (G) Each period obtained from (F). Black points indicate the minimum value in each period. (H) Periods for the analysis of slope and activity (gray shadowed box). (I) Each period obtained from (H). Black lines indicate regression lines. (J) The threshold for the detection of activity was calculated as the standard deviation in the region before Wave 1 (blue shadowed box). (K) Example of the detection of activity. The left panel indicates nonactive ROI. The right panel indicates active ROIs. Sax blue, blue, and green horizontal lines indicate thresholds of 10, 7, and 5× the standard deviation, respectively.

**Fig. S3.** Plot of the minimum value and slope of the regression line in each ROI. (A) Minimum value of each ROI in each period. (B) Slope of the regression line of each ROI in each period. (C) The mean minimum value of each ROI. (D) The mean slope of the regression line of each ROI. The data of wild‐type mice (wt) in astrocytic and neuronal ROIs (*N* = 120, 120) and *Atp1a2^+/−^* (a2) in astrocytic and neuronal ROIs (*N* = 120, 120) were plotted.

**Fig. S4.** Activity after Ca^2+^ waves in each ROI. (A) Percentage of active ROIs above the threshold of the standard deviation ×5. (B) Percentage of active ROIs above the threshold of the standard deviation ×7. (C) Percentage of active ROIs above the threshold of the standard deviation ×10. The percentage of active ROIs in *Atp1a2^+/−^* mice (a2, *N* = 120) was higher than that in wild‐type mice (wt, *N* = 120) at thresholds of the standard deviation ×5, ×7, and ×10. (D) Sum of the duration of activity above the threshold of the standard deviation ×5. (E) Sum of the duration of activity above the threshold of the standard deviation ×7. (F) Sum of the duration of activity above the threshold of the standard deviation ×10. (G) Duration of activity per the number of activities above the threshold of the standard deviation ×5. (H) Duration of activity per the number of activities above the threshold of the standard deviation ×7. (I) Duration of activity per the number of activities above the threshold of the standard deviation ×10. (D‐I) The data of wt in astrocytic and neuronal ROIs (*N* = 120, 120) and a2 in astrocytic and neuronal ROIs (*N* = 120, 120) were plotted. (J) Peak ∆*F*/*F* of activity above the threshold of the standard deviation ×5. (K) Peak ∆F/F of activity above the threshold of the standard deviation ×7. (L) Peak ∆F/F of activity above the threshold of the standard deviation ×10.

**Fig. S5.** Activity correlation within a single astrocyte by the threshold of the standard deviation ×5. (A) Upper panel. An astrocyte labeled by sulforhodamine 101. Blue boxes indicate ROIs. Bar = 10 μm. Lower panel. Representative plot of the correlation network. R in circle means ROI. (B) The mean correlations in wild‐type mouse (wt) cells (*N* = 13) and *Atp1a2^+/−^* mouse (a2) cells (*N* = 13) were plotted. Colors show individual mice. (C) The mean correlations of wt mice (*N* = 4) and a2 mice (*N* = 5) were plotted.

**Video S1**. Representative movie of imaging of the cortex at a depth of 200 μm in wild‐type mice after CSD induction. The movie shows merged images of G‐CaMP7 (green) and sulforhodamine 101 (red) fluorescence. The movie is accelerated 12 times. Time stamps are indicated in the upper right corner of the movie The size of the imaged area is 508 × 508 µm.

**Video S2.** Representative movie of imaging of the cortex at a depth of 200 μm in Atp1a2+/− mice after CSD induction. The movie shows merged images of G‐CaMP7 (green) and sulforhodamine 101 (red) fluorescence. The movie is accelerated 12 times. Time stamps are indicated in the upper right corner of the movie. The size of the imaged area is 508 x 508 µm.

**Data S1.** File containing raw data for each figure.
