# Supplementary figures and images for: Astrocytes in Atp1a2‐deficient heterozygous mice exhibit hyperactivity after induction of cortical spreading depression
Source: FEBS Open Bio. 2020 Apr 23;10(6):1031–43. doi: 10.1002/2211-5463.12848 (PMC7262908; doi:10.1002/2211-5463.12848)

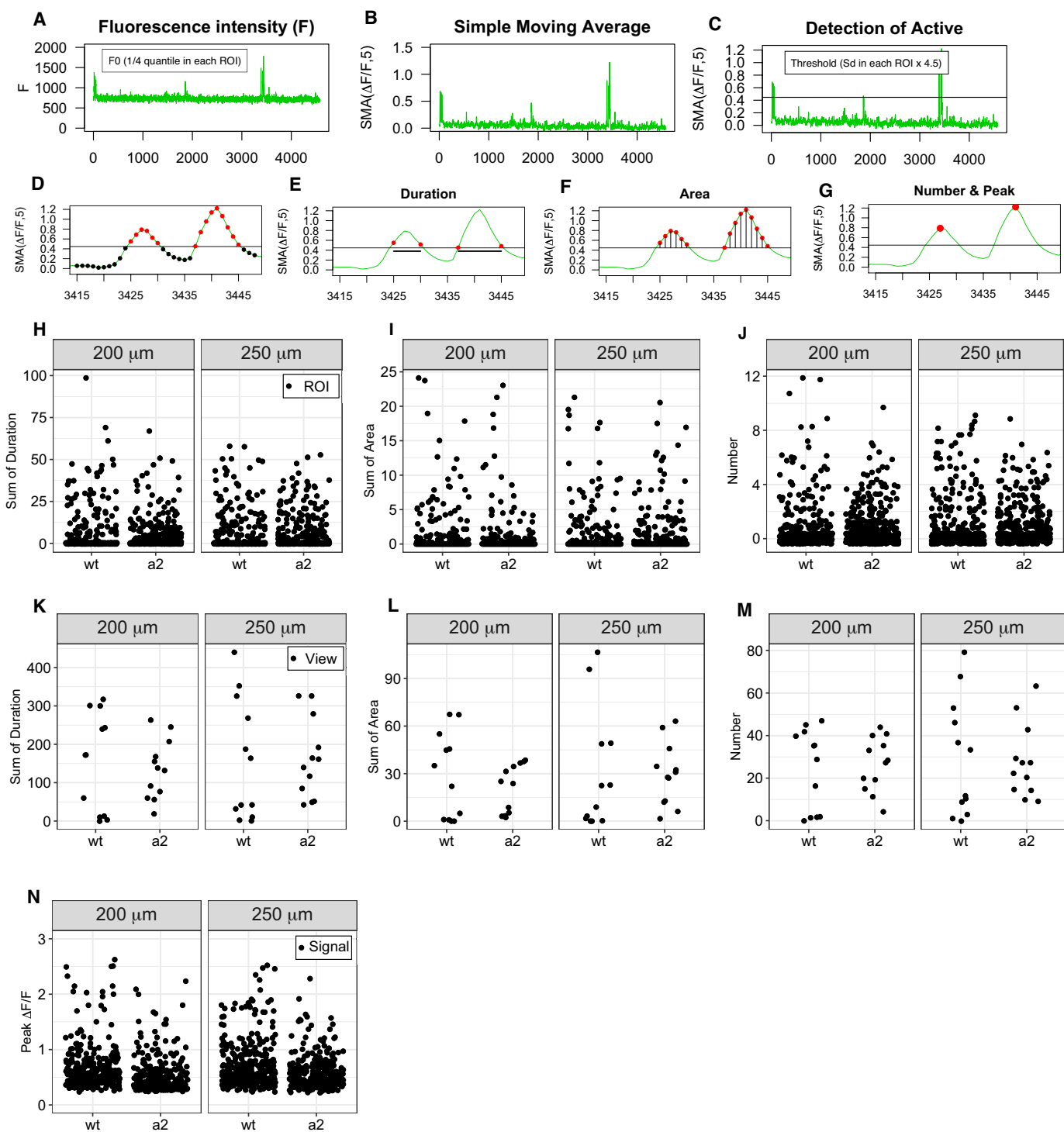

S\_Fig. 1 Sugimoto et al.

Supplement: Supplementary file 1 — Fig. S1. Spontaneous activity of neurons at depths of 200 and 250 μm in the cortex. (A) Representative data of fluorescence intensity F obtained from an ROI. First quartile data were used as F0. (B) The data applying simple moving average with a window size of 5 frames. (C) Neural activity was defined as the local maxima of ΔF/F over 4.5 standard deviations from F0 (horizontal line). (D) Detection of activity. Red points indicate activity, and black points indicate frames below the threshold. (E–G) The duration (distance between both ends of sequential red points), area (sum of ΔF/F ‐ threshold in each red point), number, and peak ΔF/F of activities were measured. (H) Sum of the duration of activity in each ROI. (I) Sum of the area of activity in each ROI. (J) Number of activities in each ROI. ROIs (N = 480) were plotted in wild‐type mice (wt) and Atp1a2+/− mice (a2) at depths of 200 and 250 μm, respectively. (K) Sum of the duration of activity in each field of view. (L) Sum of the area of activity in each field of view. (M) Number of activities in each field of view. Fields of view (N = 12) were plotted in wt and a2 at depths of 200 and 250 μm, respectively. (N) Peak ∆F/F of activities (N = 294, 351, 317, 332) in wt and a2 at depths of 200 and 250 μm, respectively. [file FEB4-10-1031-s001.pdf]

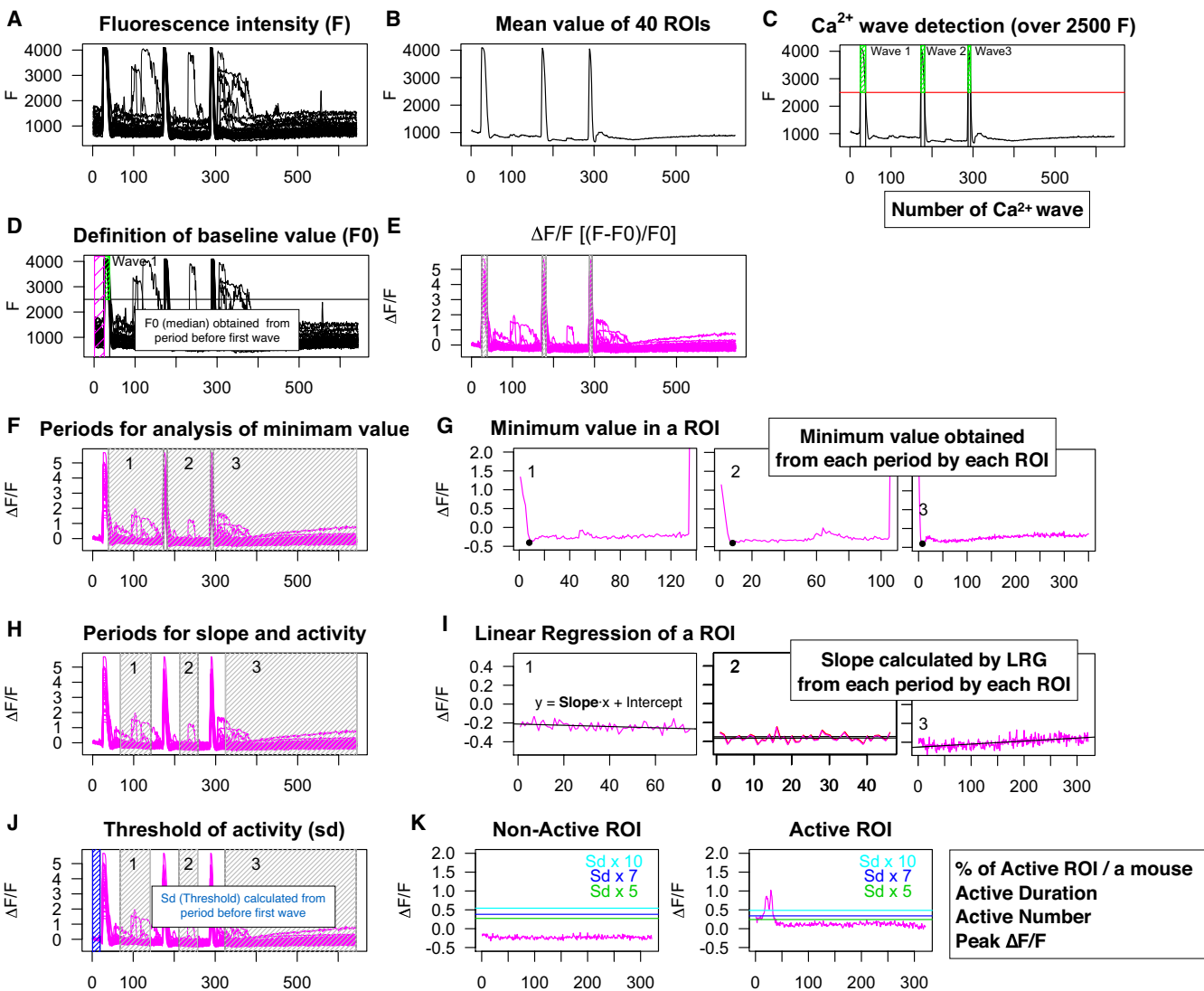

S\_Fig. 2 Sugimoto et al.

Supplement: Supplementary file 2 — Fig. S2. Analysis flow chart after CSD induction. (A) Fluorescence intensity F obtained from 20 neuronal ROIs and 20 astrocytic ROIs. (B, C) Ca2+ wave detection. (B) The mean value of fluorescence intensity across 40 ROIs. (C) Ca2+ waves (green shadowed boxes) were defined as the periods above 2500 (red horizontal line). (D) The median value of fluorescence intensity before the first wave (pink shadowed box) was defined as F0. (E) The data of ΔF/F calculated by (F ‐ F0)/F0. Gray shadowed boxes indicate periods of Ca2+ waves. (F) Periods for the analysis of the minimum value (gray shadowed boxes). (G) Each period obtained from (F). Black points indicate the minimum value in each period. (H) Periods for the analysis of slope and activity (gray shadowed box). (I) Each period obtained from (H). Black lines indicate regression lines. (J) The threshold for the detection of activity was calculated as the standard deviation in the region before Wave 1 (blue shadowed box). (K) Example of the detection of activity. The left panel indicates nonactive ROI. The right panel indicates active ROIs. Sax blue, blue, and green horizontal lines indicate thresholds of 10, 7, and 5× the standard deviation, respectively. [file FEB4-10-1031-s002.pdf]

**A**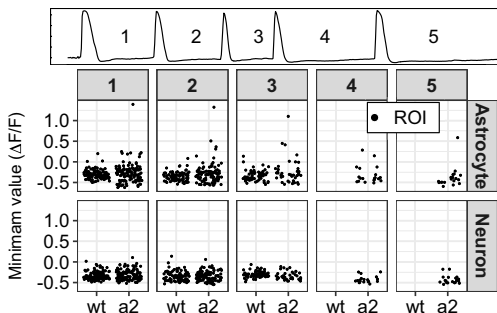**B**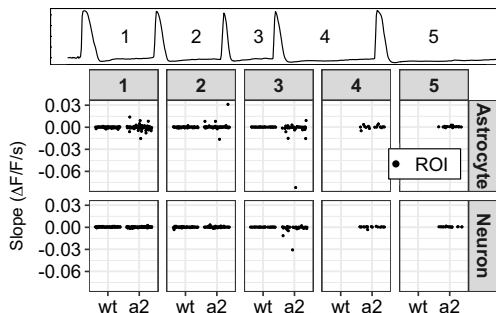**C**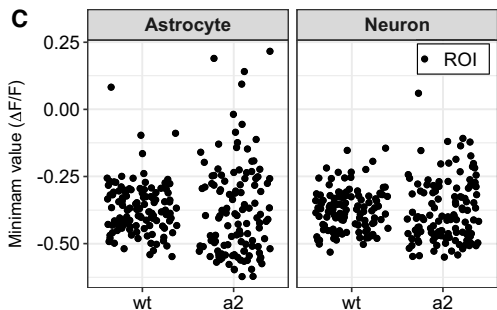**D**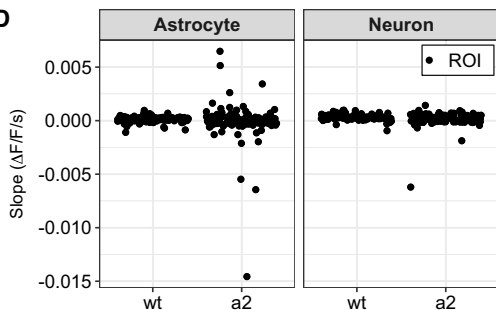

Supplement: Supplementary file 3 — Fig. S3. Plot of the minimum value and slope of the regression line in each ROI. (A) Minimum value of each ROI in each period. (B) Slope of the regression line of each ROI in each period. (C) The mean minimum value of each ROI. (D) The mean slope of the regression line of each ROI. The data of wild‐type mice (wt) in astrocytic and neuronal ROIs (N = 120, 120) and Atp1a2+/− (a2) in astrocytic and neuronal ROIs (N = 120, 120) were plotted. [file FEB4-10-1031-s003.pdf]

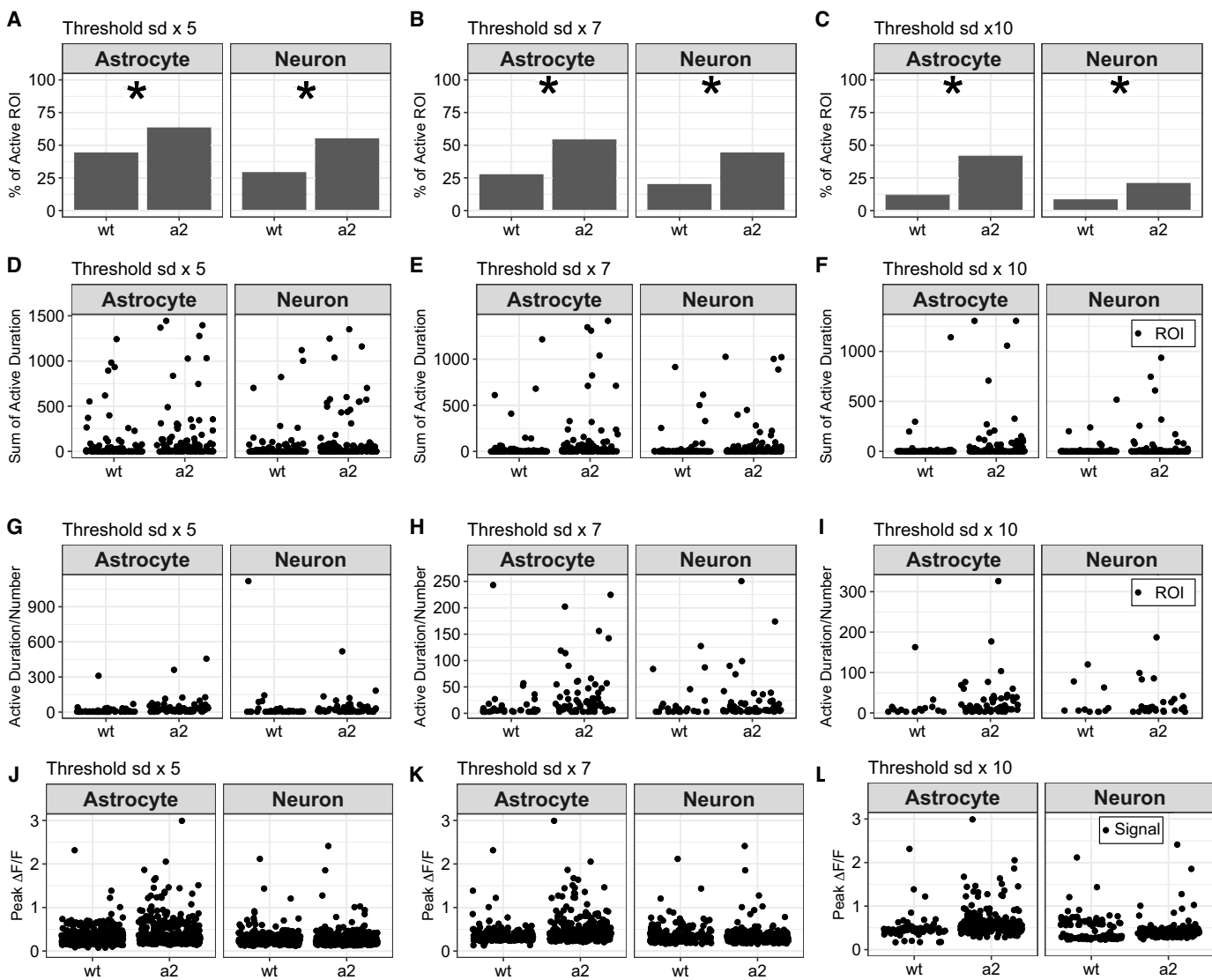

S\_Fig. 4 Sugimoto et al.

Supplement: Supplementary file 4 — Fig. S4. Activity after Ca2+ waves in each ROI. (A) Percentage of active ROIs above the threshold of the standard deviation ×5. (B) Percentage of active ROIs above the threshold of the standard deviation ×7. (C) Percentage of active ROIs above the threshold of the standard deviation ×10. The percentage of active ROIs in Atp1a2+/− mice (a2, N = 120) was higher than that in wild‐type mice (wt, N = 120) at thresholds of the standard deviation ×5, ×7, and ×10. (D) Sum of the duration of activity above the threshold of the standard deviation ×5. (E) Sum of the duration of activity above the threshold of the standard deviation ×7. (F) Sum of the duration of activity above the threshold of the standard deviation ×10. (G) Duration of activity per the number of activities above the threshold of the standard deviation ×5. (H) Duration of activity per the number of activities above the threshold of the standard deviation ×7. (I) Duration of activity per the number of activities above the threshold of the standard deviation ×10. (D‐I) The data of wt in astrocytic and neuronal ROIs (N = 120, 120) and a2 in astrocytic and neuronal ROIs (N = 120, 120) were plotted. (J) Peak ∆F/F of activity above the threshold of the standard deviation ×5. (K) Peak ∆F/F of activity above the threshold of the standard deviation ×7. (L) Peak ∆F/F of activity above the threshold of the standard deviation ×10. [file FEB4-10-1031-s004.pdf]

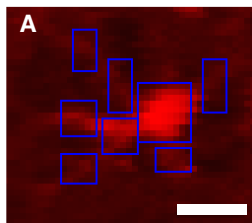

Correlation within a cell

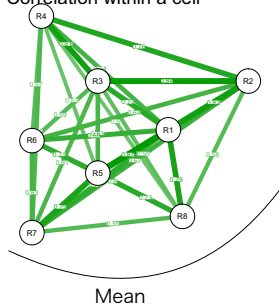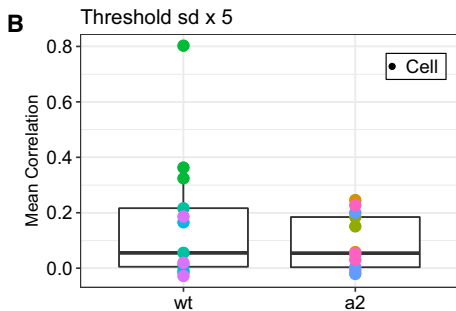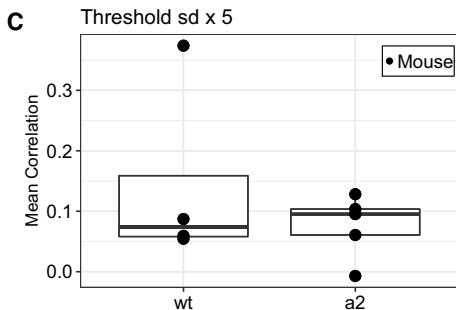

Supplement: Supplementary file 5 — Fig. S5. Activity correlation within a single astrocyte by the threshold of the standard deviation ×5. (A) Upper panel. An astrocyte labeled by sulforhodamine 101. Blue boxes indicate ROIs. Bar = 10 μm. Lower panel. Representative plot of the correlation network. R in circle means ROI. (B) The mean correlations in wild‐type mouse (wt) cells (N = 13) and Atp1a2+/− mouse (a2) cells (N = 13) were plotted. Colors show individual mice. (C) The mean correlations of wt mice (N = 4) and a2 mice (N = 5) were plotted. [file FEB4-10-1031-s005.pdf]
